# Occupational Stress and Burnout among Surgeons in Fiji

**DOI:** 10.3389/fpubh.2017.00041

**Published:** 2017-03-09

**Authors:** Rajeev Patel, Peter Huggard, Annik van Toledo

**Affiliations:** ^1^Colonial War Memorial Hospital, Suva, Fiji Islands; ^2^Faculty of Medical and Health Sciences, The University of Auckland, Auckland, New Zealand; ^3^The University of Auckland, Auckland, New Zealand

**Keywords:** stress, burnout, surgeons, psychiatric morbidity, emotional exhaustion

## Abstract

**Aim:**

This study examined the levels of occupational stress and burnout among surgeons in Fiji.

**Methods:**

A document set comprising a cover letter; a consent form; a sociodemographic and supplementary information questionnaire; the Maslach Burnout Inventory (MBI); the 12-item General Health Questionnaire (GHQ-12); the Alcohol Use Disorders Identification Test (AUDIT); and the Professional Quality of Life (ProQOL) questionnaires were provided to surgeons from three public divisional hospitals in Fiji. Thirty-six of 43 (83.7%) invited surgeons participated in the study.

**Results:**

According to their MBI scores, surgeons suffered from low (10, 27.8%), moderate (23, 63.9%), and high (3, 8.3%) levels of burnout. Comparatively, 23 (63.9%) demonstrated moderate burnout according to their ProQOL scores. Substantial psychiatric morbidity was observed in 16 (44.0%) surgeons per their GHQ-12 scores. Consumption of alcohol was noted in 29 (80.6%) surgeons, and 12 (33.4%) had AUDIT scores characterizing their alcohol use in excess of low-risk guidelines or as harmful or hazardous drinking. Surgeons of Fijian nationality showed higher MBI emotional exhaustion and depersonalization scores compared with surgeons of other nationalities. Surgeons with an awareness of the availability of counseling services at their hospitals showed low AUDIT and ProQOL burnout scores. Smokers, alcohol drinkers, and kava drinkers showed higher AUDIT scores.

**Conclusion:**

This study highlights a level of occupational stress and burnout among surgeons in Fiji and a lack of awareness of their mental and physical well-being. The authors recommend that occupational stress and burnout intervention strategies be put in place in hospitals in Fiji.

## Introduction

Occupational stress is a pattern of physiological, emotional cognitive, and behavioral responses that occur when workers are presented with work demands not matched to their knowledge, skills, or abilities and which challenge their ability to cope. Freudenberger noticed among voluntary health-care workers feelings of emotional exhaustion (EE) and withdrawal (1). Maslach further defined burnout as having three components of EE—are feelings of fatigue and of being drained by one’s work; depersonalization (DP)—are negative attitude toward and a dehumanizing treatment of one’s clients in the work place; and lack of personal accomplishment (PA)—lack of feelings of competence and achievements in one’s work with people (2).

Surgeons play an important role in health-care systems (3), and surgery is a core treatment mode in many medical interventions, including cancer, trauma, and acute-care treatments. Although surgery is considered to be a rewarding profession, it is also a field that may be associated with high levels of occupational stress and burnout (4), possibly due to the hectic and stressful nature of the role and its high physical demands and emotional challenges. The high-pressure environment in which emergency or elective surgeries are often performed; the long hours of work; continual sleep deprivation; frequent emergency calls; and intervening in life-or-death medical situations on a daily basis can eventually cause burnout in surgical staff (5, 6). Occupational burnout may impair a surgeon’s technical performance, cause mental and physical health problems, lead to medical errors, and can (in extreme circumstances) increase suicidal tendency (7).

Studies have shown that surgeons can experience high levels of job stress and low job satisfaction (8, 9). In a 10-year follow-up study on burnout and stress among surgeons, Taylor et al. demonstrated an increased level of EE and psychiatric morbidity in surgical oncologists (10). The study also found that, during an 8-year period, there was an increase in psychiatric morbidity (from 22 to 33%), an increase in burnout (from 27 to 41%), and a decrease in job satisfaction (from 68 to 65%) (10). In the UK, surgeons were shown to exhibit susceptibility to substance abuse, sharps injuries, stress and burnout, and musculoskeletal pain (11). A Korean study demonstrated that, compared with other professions, surgeons were more prone to occupational stress. Factors such as the number of duty days per week and average hours of duty were found to be significantly associated with stress among surgeons (12).

Fiji is a developing country in the South Pacific. The country’s medical system has developed surgical specialties, such as intensive care, urology, plastic surgery, neurosurgery, vascular surgery, pediatric surgery, and ophthalmology (13). Despite the prominent role played by surgeons in Fiji’s health-care system, there are no studies that exclusively examine occupational stress and burnout levels in surgeons in Fiji or the South Pacific (14). We postulate that there are high levels of burnout among surgeons in a developing country like Fiji, which was tested using a cross-sectional survey.

## Materials and Methods

### Respondents and Procedure

In Fiji, the majority of surgical services are provided by three public divisional hospitals and a private hospital, which served as the sources for the study participants. A list of all surgeons actively practicing was obtained from the Ministry of Health database. There were 43 surgeons present at the time of the study eligible who were invited to take part in the study, of which 36 (83.7%) voluntarily participated in the study—5 surgeons from Labasa Hospital, 11 from Lautoka Hospital, and 20 from Colonial War Memorial Hospital. A document set of several self-explanatory questionnaires was given to each participating surgeon. The document set comprised a cover letter; a consent form; a sociodemographic and supplementary information questionnaire; the Maslach Burnout Inventory (MBI); the 12-item General Health Questionnaire (GHQ-12); the Alcohol Use Disorders Identification Test (AUDIT); and the Professional Quality of Life (ProQOL) questionnaire. It had been previously determined that it would take approximately 30 min to complete the survey instruments. Permission to reproduce copyrighted questionnaires—the MBI Questionnaire and GHQ-12—was obtained. Ethical approval for the study was obtained from the Ministry of Health Research Committee.

### Questionnaires

#### Sociodemographic and Supplementary Sections

The sociodemographic questionnaire captured information on age, gender, ethnicity, relationship status, formal qualifications, years of training, place of work and position in the workplace, and number of years in the occupation. The supplementary section captured information on number of hours worked per week; nature of case load; hours spent in the clinic; hours spent on-call; sick leave days taken; tobacco use; alcohol and kava consumption; self-medication; quality of relationships with peers, staff, and mentors; perception of the counseling resources in the work place; and perception of level of pay.

#### MBI Questionnaire

In the medical literature, the MBI has become the gold standard for measuring burnout (15). The MBI is a 22-item questionnaire that has been shown to be a reproducible and valid survey instrument (16). It measures three components of burnout: EE, DP, and PA, each measured on a separate subscale. The study participants used a 7-point Likert scale to indicate the frequency at which they experienced certain feelings related to their work during the week prior to taking the survey (16). The EE subscale has nine items and assesses feelings of emotional overextension and work exhaustion. The DP subscale has five items that measure “an unfeeling and impersonal response toward recipients of one’s services, care, treatment, or instruction.” The PA subscale has eight items that assess feelings of occupational competence and success. The MBI norms for medical workers were developed from the responses of a sample of 1,104 American doctors and nurses (16). Scores are compiled for each subscale and are categorized by thirds in accordance with the normative distribution. High scores on the EE and DP subscales and a low score on the PA subscale were used to classify one has having burnout (16).

#### Twelve-Item General Health Questionnaire

Levels of psychiatric morbidity were assessed with the GHQ-12, a widely used mental disorder screening instrument that has previously been used with the MBI (17, 18). The 12 items represent symptoms of psychiatric morbidity. Each receives a score of 0 or 1 (based on the frequency with which the subject has experienced the symptom in the recent past), yielding a maximum score of 12 (19). Scores of 4 or higher are considered to be indicative of substantial psychiatric morbidity (19).

#### ProQOL Questionnaire

The ProQOL questionnaire is one of the most common instruments used to assess positive and negative effects experienced by persons employed in helping and caring professions (20). The ProQOL comprises three subscales: compassion satisfaction (ProQOL-CS), burnout (ProQOL-B), and compassion fatigue (ProQOL-CF). The ProQOL-CS subscale measures the level of joy and satisfaction received from one’s work. The ProQOL-B subscale measures despair and exhaustion resulting from work-related stress. The ProQOL-CF subscale measures the symptoms caused by stressful situations and the resulting secondary traumatic stress.

#### AUDIT Questionnaire

The AUDIT is a 10-question instrument developed by the World Health Organization to determine if a person’s alcohol consumption may be at a harmful level. The instrument was validated in a study using patients from six countries (21). Questions related to alcohol consumption, alcohol dependence, and alcohol-related problems are asked. A score of 8 or more in men (7 in women) indicates a strong likelihood of harmful alcohol consumption. A score of 20 or more is suggestive of alcohol dependence (21).

### Statistical Analysis

Data obtained from the MBI, GHQ-12, ProQOL, and AUDIT instruments were scored and interpreted according to criteria reported in their respective manuals. Descriptive analysis was obtained from the sociodemographic indicators section of the survey. All data were made anonymous and analyzed by an independent researcher to prevent bias and protect confidentiality. Data were analyzed using the Statistical Package for the Social Sciences (SPSS, version 11.5, Chicago, IL, USA). One-way analysis of variance (ANOVA), Student’s *t*-test, and Pearson’s product-moment correlation coefficient were used to compare the variables and various scales. Two-tailed Pearson’s correlation was used to compare between the various scales.

## Results

Table 1 shows the sociodemographic and professional characteristics of the surgeons. Thirty-two (88.9%) study participants were male. Ethnically, 12 (33.3%) study participants identified as Fijians, 8 (22.2%) as Indians, and 16 (44.4%) as “other” nationalities.

**Table 1:** **Respondents’ characteristics**.

Characteristics	Value (*n* = 36)
Age, median (range)	28 (28–56)
Gender, no. (%)	
Male	32 (88.9)
Female	4 (11.1)
Ethnicity/nationality, no. (%)	
Fijian	12 (33.3)
Indian	8 (22.3)
Others	9 (25.0)
Pacific Islanders	7 (19.4)
Relationship status, no. (%)	
Single	3 (8.2)
Married	29 (80.6)
Divorced	2 (5.6)
Cohabiting	2 (5.6)
Professional status, no. (%)	
Consultants	7 (19.5)
Registrars	
Trainee	16 (44.4)
Non-trainee	13 (36.1)
Number of years working as a surgeon, median (range)	5 (0.5–31)
Working hours per week, median (range)	60 (40–80)
Number of patients under daily care, median (range)	20 (10–30)
Number of patients seen in clinics, median (range)	20 (15–30)
Number of on-calls per month, median (range)	9 (0–30)
Smoking, no. (%)	
Yes	7 (19.4)
No	29 (80.6)
Alcohol consumption, no. (%)	
Yes	29 (80.6)
No	7 (19.4)
Kava consumption, no. (%)	
Yes	15 (41.7)
No	21 (58.3)
Awareness of counseling services provided by the hospital, no. (%)	
Yes	5 (13.9)
No	31 (86.1)
Use of counseling services within or outside the hospital, no. (%)	
Yes	0 (0.0)
No	36 (100.0)
Visit to the GP/doctor in the last year when sick, no. (%)	
Yes	5 (13.9)
No	31 (86.1)
Self-medication when sick in the last year, no. (%)	
Yes	33 (91.7)
No	3 (8.3)
Perceptions of low pay, no. (%)	
Yes	33 (91.7)
No	3 (8.3)
Perception of low/inadequate resources, no. (%)	
Yes	35 (97.2)
No	1 (2.8)

Table 2 shows the frequency of burnout, stress, and alcohol consumption among the study participants. Twenty-six (72.2%) surgeons demonstrated moderate to high levels of burnout on the MBI scale, while 23 (63.9%) demonstrated burnout on the ProQOL scale. Sixteen (44.4%) surgeons showed high levels of stress per their GHQ-12 scores.

**Table 2:** **Frequency of burnout, stress, and Alcohol Use Disorders Identification Test (AUDIT) scores**.

Characteristics	Value (*n* = 36)
**Maslach Burnout Inventory (MBI)**	
Emotional exhaustion (EE) (MBI EA) score, mean, SD	19.72 ± 6.35
Depersonalization (DP) (MBI DP) score, mean, SD	6.17 ± 2.34
Personal accomplishment (PA) (MBI PA) score, mean, SD	38.78 ± 4.30
Low burnout (low EE, low DP, and high PA), no. (%)	10 (27.8)
Moderate burnout (average EE, DP, and PA), no. (%)	23 (63.9)
High burnout (high EE, high DP, and low PA), no. (%)	3 (8.3)
**Professional Quality of Life-30 (ProQOL-30) score**	
Compassion satisfaction (ProQOL-CS) score, mean, SD	35.64 ± 8.38
Burnout score (ProQOL B), mean, SD	24.50 ± 7.03
Compassion fatigue score (ProQOL-CF), mean, SD	15.11 ± 7.02
No burnout, no. (%)	13 (36.1)
Moderate burnout, no. (%)	23 (63.9)
Severe burnout, no. (%)	0 (0.0)
**General Health Questionnaire—12 (GHQ-12) score**	
GHQ-12 score, mean, SD	3.44 ± 2.27
GHQ-12 score <4, no. (%)	20 (55.6)
GHQ-12 score ≥4, no. (%)	16 (44.4)
**AUDIT**	
Abstinence	7 (19.4)
Zone I (<7), low-risk drinking or abstinence, no. (%)	17 (47.2)
Zone II (8–15), alcohol use in excess of low-risk guidelines, no. (%)	10 (27.8)
Zone III (16–19), harmful and hazardous drinking, no. (%)	2 (5.6)
Zone IV (>20), alcohol dependence, no. (%)	0 (0.0)

Using ANOVA, the nationalities of the study participants showed a significant effect on the MBI EE [*F*_(2, 33)_ = 3.702; *p* = 0.035; *f* = 0.910] and MBI DP [*F*_(2, 33)_ = 5.357; *p* = 0.010; *f* = 0.946] scores. Bonferroni *post hoc* tests showed that surgeons of Fijian nationality (mean = 23.50, SD = 5.126) scored significantly higher on the MBI EE scale than those of the “other nationality” category (mean = 17.63, SD = 5.726; *p* = 0.041). Additionally, Bonferroni *post hoc* tests showed that surgeons of Fijian nationality (mean = 7.75, SD = 2.051) scored significantly higher than those of Indian nationality (mean = 5.00, SD = 2.673; *p* = 0.021) or those in the “other nationality” category (mean = 5.56, SD = 1.788; *p* = 0.029) on MBI DP scores.

In this study, no study participant reported accessing counseling services; however, it was found that those who were aware of the availability of counseling services (mean = 15.40, SD = 3.647) scored significantly lower on the ProQOL burnout score [*t*_(34)_ = −3.619, *p* = 0.001; *d* = 2.051] compared to those who were not aware of the availability of counseling services (mean = 25.97, SD = 6.311) on *t*-tests. It was found that surgical trainees (mean = 8.94, SD = 4.697) scored significantly higher [*t*_(34)_ = 3.770, *p* = 0.001; *d* = 1.299] than non-trainee registrars (mean = 3.10, SD = 4.553) on the AUDIT score. Furthermore, surgeons who smoked (mean 10.43, SD = 5.503) compared to those that did not smoke (mean = 4.55, SD = 4.822) scored significantly higher on the AUDIT score [*t*_(34)_ = 2.82, *p* = 0.008; *d* = 1.195].

When comparing variables on two-tailed independent-samples *T*-tests, there were no significant effects found for gender, qualification, visit to a GP, self-medication, or perceptions of low pay/inadequate resources to the MBI, GHQ-12, ProQOL, and AUDIT scales. There was a significant negative correlation between age (*r* = −0.480, *p* = 0.003) and number of years as a surgeon (*r* = −0.513, *p* = 0.001) when compared to AUDIT scores. There was a significant positive correlation between the number of hours worked per week (*r* = 0.401, *p* = 0.015) and SOPD patient load (*r* = 0.334, *p* = 0.047) and the MBI EE score. There was a significant negative correlation between the number of hours worked per week (*r* = −0.338, *p* = 0.044) and the MBI lack of PA score. The higher number of hours worked per week had a positive correlation (*r* = 0.377, *p* = 0.024) with high ProQOL burnout score (*r* = 00.512, *p* = 0.001), high GHO-12 scores, and poor ProQOL compassion satisfaction scores (*r* = 0.442, *p* = 0.007).

Age and number of years as a surgeon were significantly negatively correlated with AUDIT scores and positively correlated with GHQ-12 and MBI EE scores. The number of hours worked per week negatively correlated to the MBI PA and ProQOL-CS scores and positively correlated with ProQOL-B scores. Finally, the number of patients seen in clinics positively correlated with the MBI EE score.

When comparing questionnaires, significant correlations were found between the ProQOL-CS score and the ProQOL-B, ProQOL-CF, GHQ-12, MBI EE, and MBI DP scores. Furthermore, the ProQOL-B score significantly correlated with the ProQOL-CF and GHQ-12 scores as well as to all the MBI subscale scores. The Professional Quality of Life-30 and GHQ-12 scores also significantly correlated with all MBI subscale scores (see Table 3).

**Table 3:** **Comparison between Maslach Burnout Inventory (MBI), Professional Quality of Life (ProQOL), and 12-Item General Health Questionnaire (GHQ-12) scales**.

	ProQOL-CS	ProQOL-B	ProQOL-CF	GHQ-12	MBI emotional exhaustion (EE)	MBI depersonalization (DP)
ProQOL-B	−0.820**					
ProQOL-CF	−0.600**	0.696**				
GHQ-12	−0.404*	0.422**	0.323			
MBI EE	−0.586**	0.562**	0.491**	0.729**		
MBI DP	−0.550**	0.515**	0.471**	0.696**	0.906**	
MBI PA	0.405	−0.557**	−0.476**	−0.534**	−0.616**	−0.423**

*p < 0.05.

**p < 0.01.

*r = 0.323, p = 0.05.5*.

## Discussion

This is the first survey done among surgeons in Fiji to measure levels of occupational stress and burnout. Approximately two-thirds of the study participants surveyed showed significant levels of burnout on the MBI and ProQOL questionnaire. Further, high levels of stress were demonstrated in the study population using the GHQ-12 scale. These findings corroborate the results of Ray who showed an association between compassion fatigue, burnout, and other independent variables in a sample of frontline mental health-care professionals (22). Similar to our study, that study employed the MBI and ProQOL scales to assess burnout, compassion satisfaction, and compassion fatigue.

A major proportion of the participating surgeons had not visited a physician in the last year when sick. Also, a significant proportion of the surgeons were not aware that counseling services were offered by their hospitals and none of the participants had participated in counseling outside their hospitals. These findings illustrate that the study participants may have a lack of awareness and poor assessment of their well-being. These factors could be major barriers to taking proper steps at the right time to care for their mental and physical health (23). A high percentage of the participating surgeons had perceptions of inadequate or low levels of occupational resources and perceptions of low pay. Perceptions of low pay may lead to lower levels of job satisfaction, and these may, in turn, cause psychological stress and burnout (even in other allied medical professionals, such as nurses) (24).

It was observed in this study that surgeons in Fiji had a high workload, evident from the reported high numbers of hours of work per week and high patient load in specialty clinics. Our results are similar to those reported in a study of Korean surgeons where long hours of work, frequent duties at night, and high numbers of patients assigned per day were associated with burnout among those surgeons (12).

Another major finding in the study is that alcohol consumption was frequent in surgeons. These results corroborate the findings of Oreskovich, which demonstrated that approximately 15.4% of that study’s sample of 7,197 surgeons had AUDIT scores that were consistent with dependence on alcohol or alcohol abuse (25). Approximately 41.7% of the surgeons consumed varying amounts of kava during a week. Kava is a traditional and sociocultural drink used widely in the South Pacific and is known to have sedative, analgesic, and anti-stress properties (26).

A major concern identified in this study was that a high majority of the surgeons self-prescribed medications for illnesses during the preceding year. An early decision of the researchers was not to ask about recreational drug use as the small sample size could have led to the identification of an individual participant.

Occupational burnout is a result of job-associated stress that occurs in the long term. Surgeons may have initially viewed their occupations as being challenging and meaningful, but, in due course, found it to be unfulfilling, meaningless, and exhausting. Burnout can cause feelings of involvement and efficacy to transition into cynicism and ineffectiveness (27). This may lead to surgeons resigning, causing further human capacity shortages and increased pressure on surgical services and, thereby, perpetuating the problem.

### Study Limitations

There are some limitations to this study. Not all surgeons agreed to participate; therefore, results cannot be seen as representative of all surgeons in Fiji. However, 36 out of 43 (83.7%) surgeons did participate. Also, the small population of study participants could lead to response bias. This also reduces the power of statistical analysis and increases the risk of type II errors. To the best of our knowledge, the survey instruments had not previously been used in surveys with health professionals in Fiji. Also, no attempt was made to examine the need for adjustments in language used in the surveys or in the nature of the constructs being measured with respect to any unique cultural or ethnic differences. However, as these instruments have been used with a variety of populations, it would appear that the nature of the constructs being measured appear to be recognized across different ethic and culture boundaries. The results of the study cannot be compared to other cadres of health-care professionals as this is the first study regarding burnout in Fiji or Pacific Island country.

## Conclusion

There are several implications of this study for the health-care system of Fiji. Surgeons with a lack of awareness of counseling services and who neglect their mental and physical well-being could cause significant strain on the quality of the health-care system in the country. This suggests that there is an urgent need to devise interventions offering interactive self-assessment that could, in turn, lead to behavioral changes that improve the mental and physical well-being of the surgeons.

This is the first study of occupational stress and burnout among surgeons in Fiji. Results show similarities to previous studies having larger populations of health professionals, which report high levels of stress and burnout and reduced physical and mental health. There is a clear message about the level of distress in the population studied, and this message is important and should be acted upon to improve the well-being among surgeons in Fiji that could result in improved quality of surgical services available in the country.

## Ethics Statement

All subjects gave written informed consent in accordance with the Declaration of Helsinki. The protocol was approved by the Fiji Ministry Health Research Committee (Approval Number: FNRERC Reference Number 2009-20).

## Author Contributions

All authors list have made substantial, direct, and intellectual contribution to the work and approved it for publication.

## Conflict of Interest Statement

The authors declare that they have no financial or personal relationships that may have inappropriately influenced them in writing this paper.
